# A Poly(Acrylamide-*co*-Acrylic Acid)-Encapsulated Nitrification Inhibitor with Good Soil-Loosening, Phosphorous-Solubilizing, and Nitrogen Fixation Abilities and High-Temperature Resistance

**DOI:** 10.3390/polym17091280

**Published:** 2025-05-07

**Authors:** Hui Gao, Yuli Fu, Tianyu Wang, Meijia Liu, Jianzhen Mao, Feng Xu

**Affiliations:** 1 State Key Laboratory of Green Papermaking and Resource Recycling, Qilu University of Technology, Shandong Academy of Sciences, Jinan 250353, China; 2 Key Laboratory of Paper Science and Technology of Ministry of Education, Faculty of Light Industry, Qilu University of Technology, Shandong Academy of Sciences, Jinan 250353, China; 3 Beijing Key Laboratory of Lignocellulosic Chemistry, Beijing Forestry University, Beijing 100083, China

**Keywords:** nitrification inhibitor, 3,4-dimethyl pyrazole, soil-loosening ability, phosphorous-solubilizing ability, nitrogen fixation ability, high-temperature stability

## Abstract

3,4-dimethylpyrazole (DMPZ), when used as a nitrification inhibitor, exhibits volatility, poor thermal stability, high production costs, and limited functionality restricted to nitrogen fixation. To address these limitations and introduce novel phosphorus-solubilizing and soil-loosening abilities, herein, a poly (acrylamide-*co*-acrylic acid)-encapsulated NI (P(AA-*co*-AM)-*e*-NI) is synthesized by incorporating linear P(AM-*co*-AA) macromolecular structures into NI systems. The P(AA-*co*-AM)-*e*-NI demonstrates an obvious phase transition from a glassy state to a rubbery state, with a glass transition temperature of ~150 °C. Only 5 wt% of the weight loss occurs at 220 °C, meeting the temperature requirements of the high-tower melt granulation process (≥165 °C). The DMPZ content in P(AA-*co*-AM)-*e*-NI is 1.067 wt%, representing a 120% increase compared to our previous products (0.484 wt%). P(AA-*co*-AM)-*e*-NI can effectively reduce the abundance of ammonia-oxidizing bacteria and prolong the duration during which nitrogen fertilizers exist in the form of ammonium nitrogen. It can also cooperatively enhance the conversion of insoluble phosphorus into soluble phosphorus in the presence of ammonium nitrogen (NH_4_^+^-N). In addition, upon adding P(AA-*co*-AM)-*e*-NI into soils, soil bulk density and hardness decrease by 9.2% and 10.5%, respectively, and soil permeability increases by 10.5%, showing that it has a good soil-loosening ability and capacity to regulate the soil environment.

## 1. Introduction

Amide N fertilizers in the soil can be hydrolyzed into NH_4_^+^-N, which is readily absorbed and utilized by plant roots. Therefore, in agriculture, amide N fertilizers, like ammonium N fertilizers, are extensively employed to increase crop yields [1,2,3]. However, under the influence of ammonia-oxidizing bacteria (AOB), NH_4_^+^-N can be converted into nitrate nitrogen (NO_3_^−^-N), which is prone to loss through leaching into soil and water [4,5,6]. Research has demonstrated that NIs can effectively inhibit the activity of AOB and further slow down the transformation of the above conversion process [7,8,9,10,11], and they are therefore commonly utilized as fertilizer additives to reduce the loss of nitrogen fertilizers and prolong the efficacy period of NH_4_^+^-N. Correspondingly, the reduction in nitrogen fertilizer losses also mitigates their impact on crop quality and yield, as well as the environmental pollution of the atmosphere, surface water, and groundwater. However, currently widely used NIs, such as DMPZ [10,11,12,13,14], exhibit volatility, high production costs, poor high-temperature stability, and limited nitrogen fixation capabilities.

Volatilization and temperature resistance are improved by introducing the polyacrylic acid (PAA) macromolecular structure into NI systems, and a novel phosphorus-solubilizing ability is also given [15,16,17]. However, in previous studies, the content of DMPZ remained relatively low, and further enhancements in other functionalities are required. In addition, issues such as soil compaction, reduced air and water permeability, and severe crusting caused by excessive fertilization necessitate further resolution. Therefore, introducing the flocculating macromolecular structures of polyacrylamide (PAM) [18,19,20,21,22,23] into NI systems represents a significant challenge, aiming to endow NI with a novel soil-loosening ability while preserving its original nitrogen fixation and phosphorus-solubilizing capabilities.

In this work, by complexing high-temperature-resistant silica (H_2_SiO_3_) with DMPZ and subsequent encapsulation with P(AM-*co*-AA), a P(AA-*co*-AM)-*e*-NI with a structure of P(AM-*co*-AA)/H_2_SiO_3_·DMPZ/P(AM-*co*-AA) is synthesized. In this way, the macromolecular structures of P(AM-*co*-AA) are introduced into the NI systems, thereby endowing NI with novel soil-loosening and phosphorous-solubilizing abilities. Its resistance to high temperatures is enhanced by the complexation of H_2_SiO_3_ with DMPZ and by the encapsulation of the polymer matrix. Furthermore, the content of DMPZ in P(AA-*co*-AM)-*e*-NI has been increased by optimizing the synthesis processes and conditions. The cooperative ability of P(AA-*co*-AM)-*e*-NI to accelerate the conversion of insoluble phosphorus into soluble phosphorus in the presence of NH_4_^+^-N was also investigated. This study addresses the shortcomings of the currently widely used NI and fills the research gap regarding the simultaneous possession of high-temperature resistance, soil-loosening ability, phosphorous-solubilizing ability, and nitrogen fixing ability. It also provides innovative ideas and methods on how to develop NI.

## 2. Experimental Sections

### 2.1. Fabrication Processes

The fabrication processes of P(AA-*co*-AM)-*e*-NI, as illustrated in Figure S1, are described as follows:

(I) A total of 10 g of sodium silicate (Na_2_SiO_3_, 97%, Shanghai Macklin Biochemical Co., Ltd., Shanghai, China) was dissolved in 200 g of deionized water under magnetic stirring (C MAG HS7, KA Werke GmbH & Co. KG, Guangzhou, China) at 100 rpm for 20 min. After ensuring that the Na_2_SiO_3_ was completely dissolved, 10 g of sulfuric acid (H_2_SO_4_, 98%, Yantai Yuandong Fine Chemicals Co., Ltd., Yantai, China) was added to react with the Na_2_SiO_3_ to produce H_2_SiO_3_. Then, 20 g of acrylamide (AM, 99%, Shanghai Macklin Biochemical Co., Ltd., Shanghai, China) and 5 g of acrylic acid (AA, 99%, Tianjin Damao Chemical Reagent Factory, Tianjin, China) were injected. Subsequently, 5.6 g of self-made compound initiators was slowly introduced into the mixture, which was then magnetically stirred at a speed of 100 rpm for 30 min. The reaction temperature was lastly increased to 45 °C, where AM was co-polymerized with AA for a duration of 3.5 h.

(II) A total of 100 g of the solution obtained from step (I) was redissolved in 100 g of deionized water. To ensure complete dissolution, the magnetic stirring speed was set at 250 r/min. After complete dissolution, equal masses of Na_2_SiO_3_ and H_2_SO_4_ were added again, and the reaction between Na_2_SiO_3_ and H_2_SO_4_ was carried out at room temperature for 30 min. Subsequently, 30 g of AA and 10 g of DMPZ (Xiya Reagent Co., Ltd., Linyi, China) were successively added and magnetically stirred at 250 rpm for 10 min. Once DMPZ was fully dissolved, 4.5 g of the self-made compound initiator was slowly injected to induce the copolymerization of P(AM-*co*-AA) with AA and the complexation of H_2_SiO_3_ with DMPZ. Finally, P(AA-*co*-AM)-*e*-NI was obtained after reacting at 75 °C for 4 h.

Herein, the compound initiator was prepared in our laboratory according to ref. [16].

The powder samples, which were prepared by precipitating P(AA-*co*-AM)-*e*-NI in anhydrous ethanol (99.5%, Shanghai Macklin Biochemical Co., Ltd., Shanghai, China), followed by drying at 70 °C for 1 h and subsequent grinding, were used to test the glass transition temperature (*T*_g_), temperature resistance, and chemical structure of P(AA-*co*-AM)-*e*-NI.

### 2.2. Methods

To obtain the appearance time range of the DMPZ absorption peak, a high-performance liquid chromatograph (HPLC, VERTEX-70, Bruker, Ettlingen, Germany) equipped with a C18 column was first used to test the HPLC images of DMPP standard aqueous solutions with different concentrations and then the HPLC image of P(AA-*co*-AM)-*e*-NI aqueous solution with a concentration of 17.18025 g/mL. Based on the relationship between the DMPZ absorption peak area and the concentration of the DMPP standard aqueous solution, a linear standard equation was obtained between them to calculate the content of DMPZ complexed in P(AA-*co*-AM)-*e*-NI. During the test, the C18 column temperature was set to 30 °C, the test wavelength was 224 nm, the flow rate of acetonitrile/0.1‰ phosphoric acid (flow phase) was controlled at 0.8 mL/min, and the injection volumes of both DMPP and P(AA-*co*-AM)-*e*-NI aqueous solutions were controlled at 5 μL.

Chemical structures of P(AA-*co*-AM)-*e*-NI at a wavenumber range between 4000 and 500 cm^−1^ were characterized by Fourier transform infrared spectroscopy (FTIR, Vertex-70, Bruker, Karlsruhe, Germany). The resolution and number of scans during FTIR tests were set to 4 cm^−1^ and 32, respectively. The components in P(AA-*co*-AM)-*e*-NI among the 2*θ* rang of 5~90° were also characterized using a high-resolution X-ray diffractometer (XRD, D8 Advance, Bruker, Karlsruhe, Germany). The emission source employed Cu Kα radiation with a wavelength of 1.5405 nm. During the XRD tests, the scanning speed was set to 0.4 s per step, while the tube voltage and current were maintained at 40 kV and 40 mA, respectively.

The temperature resistance of P(AA-*co*-AM)-*e*-NI at 25~700 °C was measured using thermogravimetric analysis (TGA, 18 TGAQ50/DSAQ20, New Castle County, DE, USA). The phase transition behavior and *T*_g_ of P(AA-*co*-AM)-*e*-NI at 25~200 °C were characterized by using a differential scanning calorimeter (DSC, TGAQ50/DSAQ20, New Castle County, DE, USA). During both DSC and TGA tests, the temperature gradient was controlled at 5 °C/min, and dry nitrogen was utilized as a protective gas. Herein, *T*_g_ is defined as the midpoint of the step-like increase in *C*_p_, while the decomposition temperature (*T*_d_) was determined as the temperature corresponding to 5% weight loss.

The phosphorus-solubilizing capability of P(AA-*co*-AM)-*e*-NI was evaluated by assessing its ability to convert insoluble phosphates into soluble phosphorus. Specifically, a turbid insoluble phosphate solution was prepared by mixing 5 mL of 0.05 g/mL calcium nitrate solution and 5 mL of an orthophosphate solution (pH = 7.8) in 200 mL of deionized water. Subsequently, P(AA-*co*-AM)-*e*-NI was added into the above suspension to observe whether clarification occurred. The entire phosphorus-solubilizing process was recorded using a camera. Additionally, the influence of the NH_4_^+^-N content on the time and degree of clarification of the insoluble phosphate turbid solution was investigated.

The soil-loosening ability of P(AA-*co*-AM)-*e*-NI was demonstrated by comparing the formation of soil aggregates in soil aqueous solution before and after its addition. The experimental protocol consisted of adding 50 g of finely sieved soil and 200 mL of deionized water into two graduated cylinders, followed by vigorous shaking to create a homogeneous soil suspension. Then, 2 mL of P(AA-*co*-AM)-*e*-NI was put into one cylinder, and the other served as a control group without the additive. To observe changes in soil particle behavior, including settling time, the final settling height of soil particles, and aggregate formation, both cylinders were shaken again and placed side by side.

Changes in soil density and compactness at a depth of 0~10 cm after the application of 15 g of P(AA-*co*-AM)-*e*-NI were quantitatively assessed using the cutting ring method and a soil compaction tester (TJSD-750-II, Zhejiang Top Cloud-Agri Technology Co., Ltd., Hangzhou, China). Soil porosity was calculated from bulk density and soil-specific gravity (2.65 g/cm^3^). The test was conducted on a 10 m^2^ plot located in two adjacent areas on the campus of Qilu University of Technology, Shandong Academy of Sciences.

The nitrogen fixation ability of P(AA-*co*-AM)-*e*-NI was investigated by detecting the changes in soil NH_4_^+^-N, NO_2_^−^-N and NO_3_^−^-N contents, as well as AOB abundance under different incubation times. Three treatments were prepared, 0 g of P(AA-*co*-AM)-*e*-NI (CK), 0.4573 g of P(AA-*co*-AM)-*e*-NI (test group), and 0.0503 g of DMPP (comparison group), each dissolved in 100 mL of deionized water and mixed with 1.0714 g of urea dissolved in 100 mL of deionized water. These mixtures were evenly sprayed onto 25 g of dry corn–wheat rotation soil. All groups were cultured under controlled conditions at 25 °C with 20% relative humidity for 0 h, 16 h, 32 h, 48 h, 72 h, 120 h, 168 h, 240 h, 360 h, and 600 h, respectively. Changes in the contents of soil NH_4_^+^-N, NO_2_^−^-N and NO_3_^−^-N were quantified using destructive sampling methods combined with a continuous flow analytical system. The copy numbers of AOB at 168 h, 240 h, 360 h, and 600 h were determined by real-time PCR analysis. It is worth noting that the DMPZ content in the total amount of P(AA-*co*-AM)-*e*-NI is comparable to that in the total amount of DMPP.

## 3. Results and Discussion

### 3.1. Content of DMPZ in P(AA-co-AM)-e-NI

**Figure 1:**
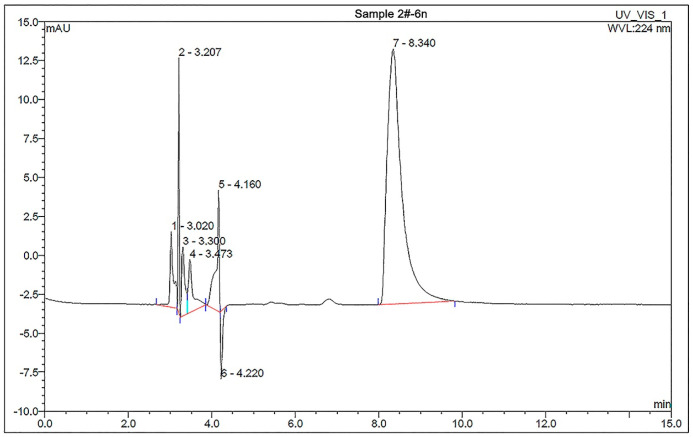
HPLC figure of P(AA-*co*-AM)-*e*-NI with a concentration of 17.18025 g/mL.

HPLC figure of the DMPP standard sample with different aqueous solution concentrations is shown in Figure S2. It can be seen that the absorption peak of DMPZ appears between 8 and 10 min. The linear standard equation (y = 2.6733x − 0.8473, R^2^ = 0.9994), as demonstrated in Figure S3, is also obtained by fitting the concentrations of DMPP standard aqueous solutions with the integral areas of the DMPZ absorption peaks. The HPLC picture of P(AA-*co*-AM)-*e*-NI with an aqueous solution concentration of 17.18025 μg/mL is shown in Figure 1.

Although some small miscellaneous peaks of other components appear in the 2~5 min time range, the DMPZ absorption peak, with an integral area of 18.8736, still appears significantly in the 8~10 min time range. This confirms the successful complexation of DMPZ with H_2_SiO_3_, indicating that P(AA-*co*-AM)-*e*-NI contains DMPZ. Using Equations (S1) and (S2) and the linear standard linear equation (y = 2.6733x − 0.8473, R^2^ = 0.9994), The calculated DMPZ content in P(AA-*co*-AM)-*e*-NI is 1.067 wt%, which is 120% higher than that in our previous product (0.484 wt%) [16]. The higher the content of DMPZ in P(AA-*co*-AM)-*e*-NI, the better the inhibitory effect on AOB activity, thus significantly inhibiting the conversion of NH_4_^+^-N to NO_3_^−^-N. As a result, the utilization rate of nitrogen fertilizers can be effectively improved, and crop yields can also be significantly increased, even with reduced application amounts of nitrogen fertilizers.

### 3.2. Chemical Structures

**Figure 2:**
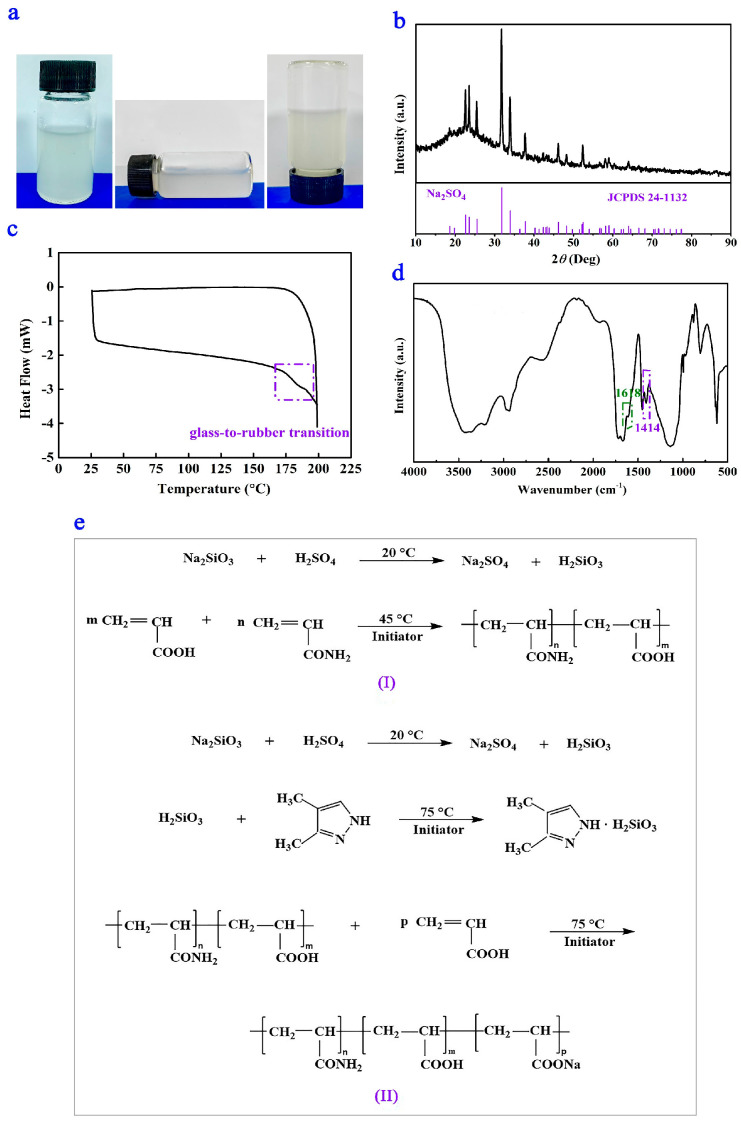
Practical product (**a**), XRD curve (**b**), DSC curve (**c**), FTIR spectrum (**d**), and chemical reactions (**e**) during P(AA-co-AM)-e-NI synthesis.

The actual product is demonstrated in Figure 2a. The XRD and DSC curves of P(AA-*co*-AM)-*e*-NI are shown in Figure 2b and Figure 2c, respectively. During the synthesis of P(AA-*co*-AM)-*e*-NI, the formation of sodium sulfate (Na_2_SO_4_) was observed. Furthermore, P(AA-*co*-AM)-*e*-NI exhibits an obvious phase transition from a glassy state to a rubbery state. The FTIR spectrum of P(AA-*co*-AM)-*e*-NI is shown in Figure 2d. In this figure, the absorption peaks corresponding to SO_4_^2−^ at 1138 cm^−1^ and 619 cm^−1^, as well as the absorption peak of Si-O-H at 967 cm^−1^, are identified. These findings confirm the successful production of H_2_SiO_3_ and Na_2_SO_4_, which aligns with the results obtained from XRD analysis. In addition, no absorption peaks are detected for the =CH_2_ and =CH groups present in both AA and AM at 983 cm^−1^ and 818 cm^−1^, respectively. This indicates that AM is completely copolymerized with AA. This result is further supported by the observation that the *T*_g_ of P(AA-*co*-AM)-*e*-NI (~150 °C) lies between those of pure PAA (~80 °C) and PAM (~180 °C).

New absorption peaks at 1618 cm^−1^ and 1414 cm^−1^, which are absent in AA, AM, and DMPZ, are found. It has been proved that the asymmetric and symmetric stretching vibration peaks of COO^−^ in COONa appear at 1546 cm^−1^ and 1405 cm^−1^, respectively [15,16]. However, these peaks are prone to redshift due to the hydrogen bonding interactions [24,25,26]. Therefore, the absorption peaks observed at 1618 cm^−^^1^ and 1414 cm^−^^1^ correspond to the asymmetric and symmetric stretching vibrations of COO^−^ in COONa, respectively. These vibrations result from hydrogen bonding interactions between the NH_2_ and COOH groups within the P(AA-*co*-AM) macromolecular chain. Additionally, the absorption peak at 1722 cm^−^^1^ is attributed to the stretching vibration of non-dissociated COOH groups [27].

Given that the DMPZ content in P(AA-*co*-AM)-*e*-NI is lower (1.067 wt%) than the theoretical value (3.78 wt%), and considering the presence of COOH and NH_2_ in the side chains of P(AA-*co*-AM)-*e*-NI, it is reasonable to conclude that the absorption peak at 3433 cm^−^^1^ results from the combined stretching vibrations of the NH group on the DMPZ pyrazole ring, the COOH and NH_2_ groups in the side chains of P(AA-*co*-AM)-*e*-NI, as well as the OH group in H_2_SiO_3_. Despite the weak spectral information regarding the pyrazole ring structure of DMPZ due to its low concentration [28], Section 3.1 and Section 3.4 have comprehensively validated the presence of DMPZ molecules in P(AA-*co*-AM)-*e*-NI and their efficacy in inhibiting the transformation of nitrogen elements in soil. Based on this analysis, the chemical reactions involved in the synthesis of P(AA-*co*-AM)-*e*-NI are shown in Figure 2e.

### 3.3. High-Temperature Resistance

**Figure 3:**
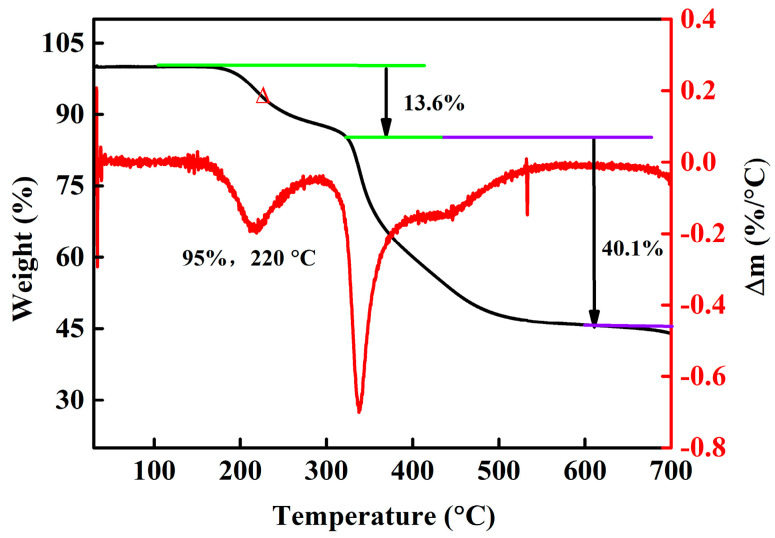
TGA and DTG curves of P(AA-*co*-AM)-*e*-NI.

The weight losses of P(AA-*co*-AM)-*e*-NI between 25 and 700 °C are illustrated in Figure 3. P(AA-*co*-AM)-*e*-NI exhibits two primary thermal degradation stages, which occur between 100~300 °C and 300~550 °C, respectively. The corresponding losses are 13.6 wt% and 40.1 wt%, respectively. At 700 °C, (AA-*co*-AM)-*e*-NI exhibits a final carbon residue of 44.0 wt%, with a *T*_d_ of ~220 °C. This *T_d_* is significantly higher than both the volatilization temperature of pure DMPZ (<50 °C) and the value reported for our earlier products (216 °C) [16]. The above results show that P(AA-*co*-AM)-*e*-NI can withstand high temperatures, fully satisfying the temperature requirements (≥165 °C) for the high-tower melting granulation process. Consequently, it can be employed directly as a functional additive in this process to produce compound fertilizers with superior comprehensive properties. In addition, P(AA-*co*-AM)-*e*-NI demonstrates versatility as a functional additive in liquid and water-soluble fertilizers, which exhibit no dependency on high-temperature processing.

### 3.4. Nitrogen Fixation Ability

**Figure 4:**
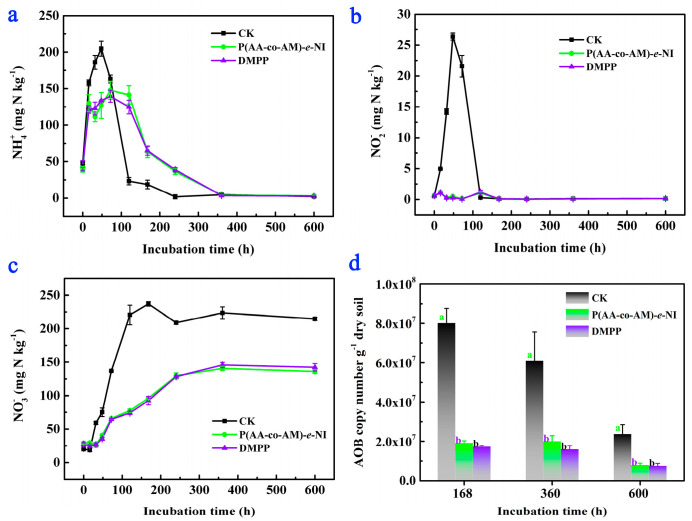
Changes in soil NH_4_^+^-N (**a**), NO_3_^−^-N (**b**), and NO_2_^−^-N (**c**) contents and AOB copy numbers (**d**) under different incubation times. Different lowercase letters represent statistically significant differences among treatments according to the LSD multiple range test (*p* < 0.05).

The variations in soil NH_4_^+^-N content under different incubation times are illustrated in Figure 4a. The NH_4_^+^-N content in the blank group (CK), test group (P(AA-*co*-AM)-*e*-NI), and comparison group (DMPP) exhibited a consistent pattern: an initial increase, followed by a gradual decline, ultimately stabilizing at approximately 3 mg·N·kg^−^^1^. Specifically, the NH_4_^+^-N content in CK reached its maximum value of 204.8 mg·N·kg^−1^ at 48 h and then rapidly dropped to 25 mg·N·kg^−1^ by 120 h and further stabilized at 3 mg·N·kg^−1^ after 120 h. By contrast, the NH_4_^+^-N content in the other two groups increased to the maximum value at 72 h (163.1 mg·N·kg^−1^ for P(AA-*co*-AM)-*e*-NI and 139.5 mg·N·kg^−1^ for DMPP), followed by a gradual reduction to ~3 mg·N·kg^−1^ by 360 h. These results indicate that P(AA-*co*-AM)-*e*-NI significantly extends the retention duration of NH_4_^+^-N in soil.

Changes in soil NO_2_^−^-N accumulation amounts across different incubation periods are illustrated in Figure 4b. The NO_2_^−^-N accumulation amount in CK remains consistently higher than those in the other two groups and reaches its maximum value of 26.4 mg·N·kg^−1^ at 48 h. However, the NO_2_^−^-N accumulation amount in the test and comparison groups reach their respective maximum values (~1.22 mg·N·kg^−1^) at 120 h, which are considerably lower than that in CK. Changes in soil NO_3_^−^-N contents across different incubation periods are illustrated in Figure 4c. The NO_3_^−^-N contents in the CK group remain consistently higher than those in the other two groups throughout the experimental period, with nitrification rates of NH_4_^+^-N to NO_3_^−^-N maintained at near-identical levels by 168 h. These results demonstrate that P(AA-*co*-AM)-*e*-NI effectively reduces NO_2_^−^-N accumulation, slows the conversion of NH_4_^+^-N to NO_3_^−^-N, and extends the retention period of NH_4_^+^-N in soil.

The AOB copy numbers across the three experimental groups at 168 h, 360 h, and 600 h are presented in Figure 4d. The AOB copy numbers in the CK group are 8.01 × 10^7^, 6.09 × 10^7^, and 2.37 × 10^7^ copies·g^−1^, respectively. The AOB copy numbers in the test group are 1.91 × 10^7^, 2.00 × 10^7^, 7.96 × 10^6^ copies·g^−1^, respectively. The AOB copy numbers in the comparison group are 1.74 × 10^7^, 1.60 × 10^7^, and 7.54 × 10^6^ copies·g^−1^, respectively. Statistical analysis revealed that the CK group maintains significantly higher AOB copy numbers than the other two groups (*p* < 0.05), demonstrating that P(AA-*co*-AM)-*e*-NI has a notable inhibitory effect on AOB activity. It is noteworthy that both P(AA-*co*-AM)-*e*-NI and DMPP contain DMPZ as their inhibitory component, and the DMPZ content is identical in the test and comparison groups. Consequently, no statistically significant differences are observed in soil NH_4_^+^-N, NO_3_^−^-N, and NO_2_^−^-N contents, nor in AOB copy numbers, between these two groups at different incubation times.

### 3.5. Phosphorous-Solubilizing Capacity

Movie S1 demonstrates the phosphorous-solubilizing process of P(AA-*co*-AM)-*e*-NI. The dose-dependent effects on phosphorous-solubilizing ability are illustrated in Table 1. When the dosage of P(AA-*co*-AM)-*e*-NI exceeds 1 mL, the white insoluble phosphate precipitations disappear, and the turbid solution becomes clear. This transformation is likely driven by the ionic complexation of carboxyl groups on the P(AA-*co*-AM) molecular chains with metal ions in the phosphate precipitates. Subsequent encapsulation of these complexes within the polymer matrix inhibits their reinteraction with phosphate ions. In addition, the solubilizing time of phosphorus initially decreases and subsequently increases with an increase in the dosage of P(AA-*co*-AM)-*e*-NI. This phenomenon can be attributed to the fact that a higher dosage of viscous P(AA-*co*-AM)-*e*-NI leads to a prolonged dissolution time in water, thereby extending the contact and reaction time with insoluble phosphates. Furthermore, we discovered that the dosage of P(AA-*co*-AM)-*e*-NI required for turbidity solution clarification is reduced to 0.1 mL, and the time for the turbidity clarification is shortened to 9 s after adding 0.5 g of NH_4_^+^-N into the white turbid solution. This indicates that P(AA-*co*-AM)-*e*-NI can act in a cooperative manner to promote the conversion of insoluble phosphorus into soluble phosphorus.

### 3.6. Soil-Loosening Capacity

**Figure 5:**
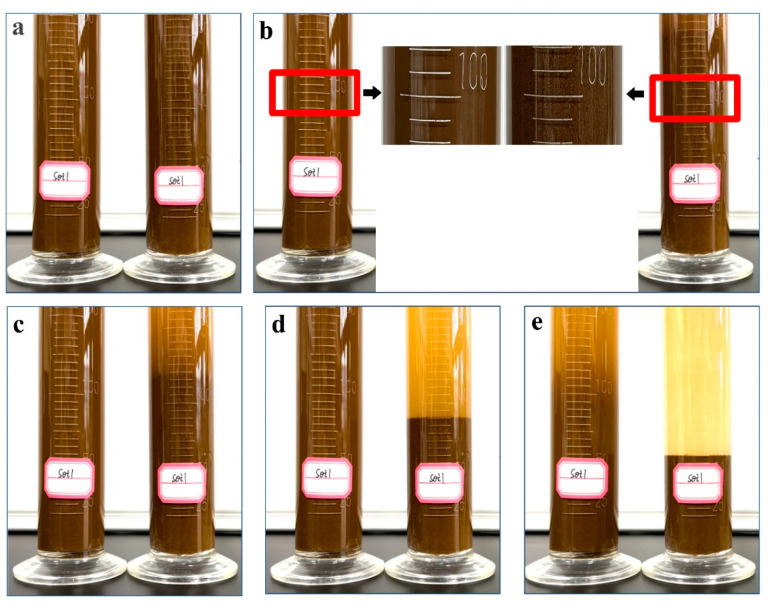
Initial formation state (**a**), intermediate settlement state (**b**–**d**), and final settlement state (**e**) of soil aggregates generated by adding P(AA-*co*-AM)-*e*-NI into soil aqueous solution.

The soil-loosening capacity of P(AA-*co*-AM)-*e*-NI is shown in Movie S2 and Figure 5. Upon injecting P(AA-*co*-AM)-*e*-NI into a soil aqueous suspension, rapid soil aggregate formation occurs, as captured in Figure 5a. The newly formed aggregates undergo sequential phase separation, with the denser aggregates precipitating first. After settling for 168 s, as illustrated in Figure 5a–d, the soil aggregates have been completely settled to the bottom of containers. The final height of the settled soil is 23.1% higher than the initial addition of fine soil, as illustrated in Figure 5e. In contrast, the fine soils in the comparison group began to settle in a discernible manner at 168 s. The preceding analysis results demonstrate that P(AA-*co*-AM)-*e*-NI not only exhibits excellent nitrogen fixation and phosphate-solubilizing abilities but also exhibits good soil-loosening properties.

The results of the soil density, porosity, and hardness tests are illustrated in Table 2. Following the incorporation of 15 g of P(AA-*co*-AM)-*e*-NI into 10 m^2^ of soil, soil density obviously decreases from 1.41 ± 0.06a g/cm^3^ to 1.28 ± 0.05a g/cm^3^. Similarly, soil hardness reduces from 419 ± 9a N/cm^2^ to 375 ± 8a N/cm^2^, while soil porosity increases from 46.8% to 51.7%. This corresponds to a 9.2% reduction in soil density, a 10.5% decrease in compactness, and a 10.5% increase in porosity. P(AA-*co*-AM)-*e*-NI is capable of promoting the formation of soil aggregates, thereby reducing soil density, increasing soil porosity, regulating the soil microenvironment, and promoting the healthy growth and development of plants.

## 4. Conclusions

In summary, a high-temperature-resistant and multifunctional soil amendment material, namely P(AA-*co*-AM)-*e*-NI, featuring an encapsulation structure of P(AA-*co*-AM)/H_2_SiO_3_·DMPZ/P(AA-*co*-AM), has been successfully synthesized. By regulating the structures and optimizing the synthesis processes, the DMPZ content (1.067 wt%) in P(AA-*co*-AM)-*e*-NI has been significantly enhanced. The temperature resistance (*T*_g_: ~150 °C, *T*_d_: ~220 °C) of P(AA-*co*-AM)-*e*-NI has also been markedly improved, and a novel soil-loosening ability has been successfully endowed. Furthermore, P(AA-*co*-AM)-*e*-NI not only inhibits the conversion of NH_4_^+^-N to NO_3_^−^-N and AOB activity but also catalyzes the conversion of insoluble phosphorus into soluble phosphorus in a cooperative manner. Therefore, when used as a nitrogen-phosphorus synergist and high-efficiency fertilizer additive, the high-temperature resistant and multifunctional P(AA-*co*-AM)-*e*-NI can regulate the soil environment, mitigate environmental pollution, improve crop yields and quality, and reduce production costs by 10^1^–10^2^ RMB per kilogram.

## Figures and Tables

**Table 1:** The dose-dependent effects on phosphorous-solubilizing ability.

	**Dosage (mL)**	0.1	0.5	1.0	2.0	3.0	5.0
**Solubilizing Ability**							
Degree		turbidity	turbidity	clarification	clarification	clarification	clarification
Time (s)		∞	∞	10	7	11	15

**Table 2:** Effects of P(AA-*co*-AM)-*e*-NI on soil density, compactness and porosity.

Sample	Density (g/cm^3^)	Compactness (N/cm^2^)	Porosity (%)
Without P(AA-*co*-AM)-*e*-NI	1.41 ± 0.06 a	419 ± 9 a	46.8%
With P(AA-*co*-AM)-*e*-NI	1.28 ± 0.05 a	375 ± 8 a	51.7%

## Data Availability

The original contributions presented in this study are included in the article/Supplementary Material. Further inquiries can be directed to the corresponding authors.

## Supplementary Materials

The supporting information for this study is available for download at https://www.mdpi.com/article/10.3390/polym17091280/s1, Figure S1: Illustration of the fabrication processes involved in the synthesis of the novel P(AA-*co*-AM)-*e*-NI; Figure S2: High-Performance Liquid Chromatography (HPLC) profiles of the DMPP standard sample at concentrations of (a) 51.60 µg/mL, (b) 102.04 µg/mL, and (c) 130.76 µg/mL; Figure S3: A plot depicting the linear relationship between the concentration of the DMPP standard aqueous solution and the integrated area of the DMPZ absorption peak; Figure S4: Changes in soil particles in soil aqueous solution after adding P(AA-*co*-AM)-*e*-NI; Equations (S1) and (S2): Formula for calculating the mass concentration ω of DMPZ in P(AA-*co*-AM)-*e*-NI; Movie S1: The phosphorous-solubilizing process of P(AA-*co*-AM)-*e*-NI; Movie S2: The soil-loosening process of P(AA-*co*-AM)-*e*-NI.
