# Supplementary material for: A Poly(Acrylamide-co-Acrylic Acid)-Encapsulated Nitrification Inhibitor with Good Soil-Loosening, Phosphorous-Solubilizing, and Nitrogen Fixation Abilities and High-Temperature Resistance
Source: Polymers (Basel). 2025 May 7;17(9):1280. doi: 10.3390/polym17091280 (PMC12073658; doi:10.3390/polym17091280)
Supplement: Supplementary file 1 [file polymers-17-01280-s001.zip › Supporting Information.pdf]

Supporting Information

A Poly(Acrylamide-*co*-Acrylic Acid)-Encapsulated Nitrification Inhibitor with Good Soil-Loosening, Phosphorous-Solubilizing, and Nitrogen Fixation Abilities and High-Temperature Resistance

Hui Gao [1,2,*], Yuli Fu [1], Tianyu Wang [1], Meijia Liu [1,2], Jianzhen Mao [1,2] and Feng Xu [1,3,*]

[1] State Key Laboratory of Green Papermaking and Resource Recycling, Qilu University of Technology, Shandong Academy of Sciences, Jinan 250353, China
[2] Key Laboratory of Paper Science and Technology of Ministry of Education, Faculty of Light Industry, Qilu University of Technology, Shandong Academy of Sciences, Jinan 250353, China
[3] Beijing Key Laboratory of Lignocellulosic Chemistry, Beijing Forestry University, Beijing 100083, China
* Correspondence: gaohui@qlu.edu.cn (H.G.); xfx315@163.com (F.X.)

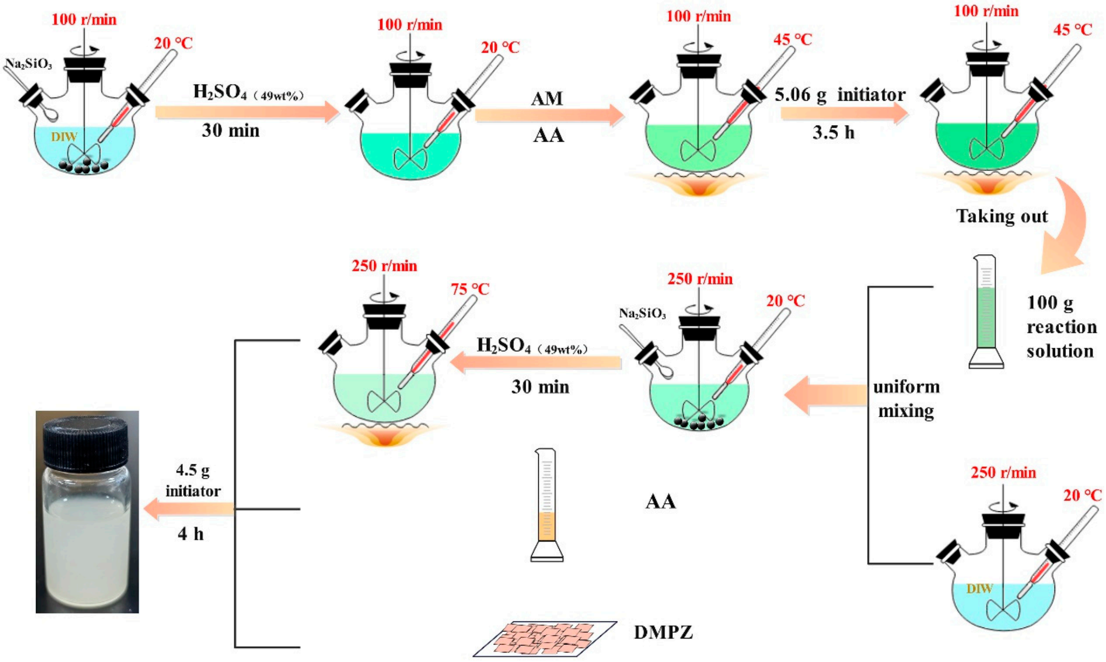

Figure S1. Illustration of the fabrication processes involved in the synthesis of the novel P(AA-*co*-AM)-*e*-NI.

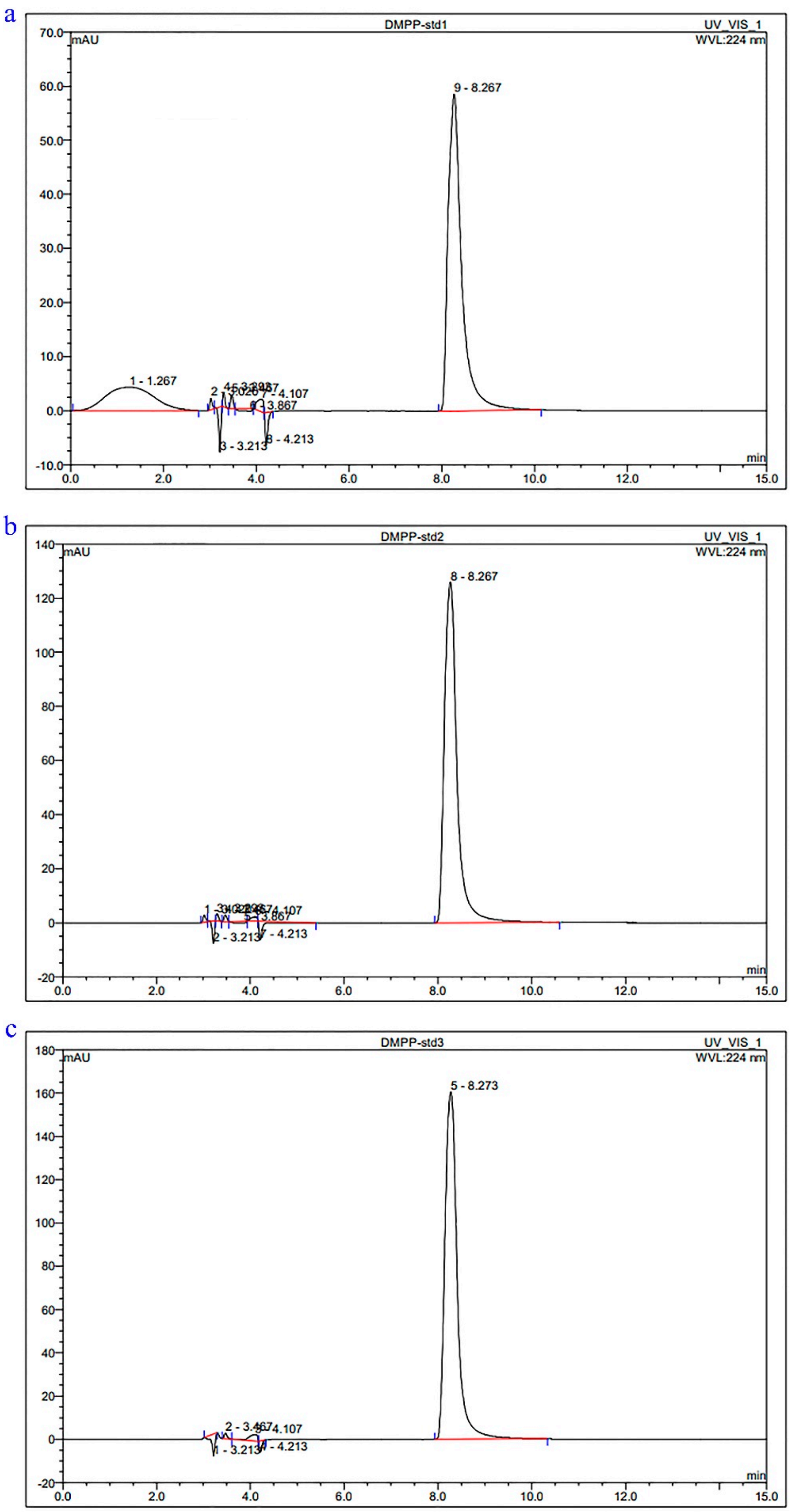

Figure S2. High-Performance Liquid Chromatography (HPLC) profiles of the DMPP standard sample at concentrations of (a) 51.60 µg/mL, (b) 102.04 µg/mL, and (c) 130.76 µg/mL.

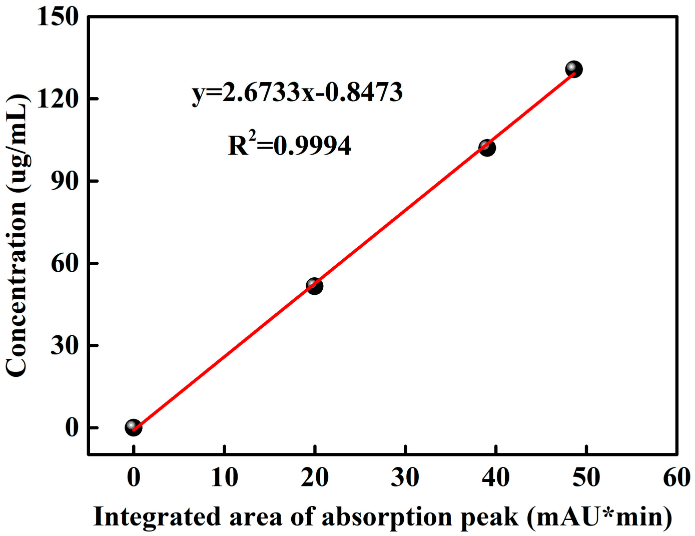

Figure S3. A plot depicting the linear relationship between the concentration of the DMPP standard aqueous solution and the integrated area of the DMPZ absorption peak.

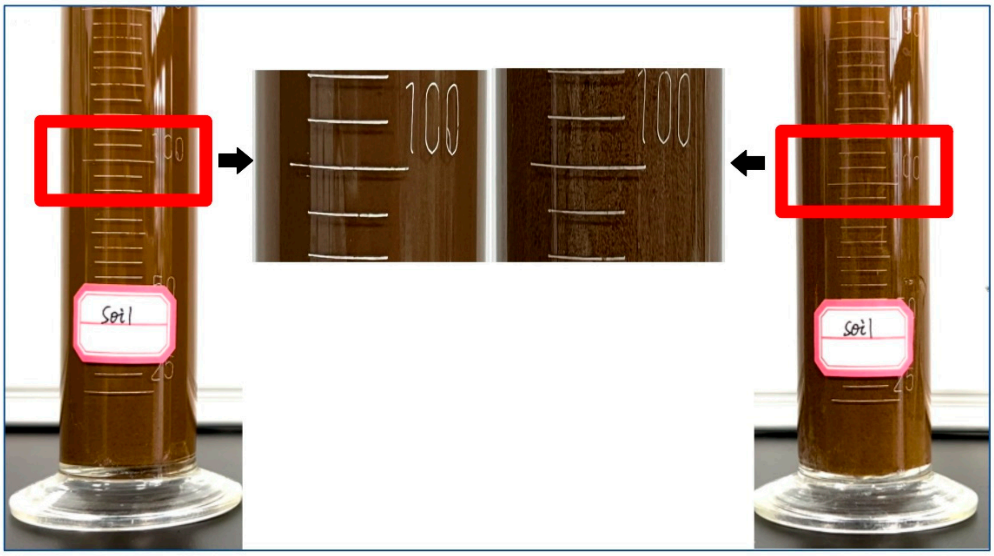

Figure S4. Changes of soil particles in soil aqueous solution after adding P(AA-*co*-AM)-*e*-NI.

Equations:

$$\omega = \frac{\rho V D \times 10^{-3}}{m \times 10^{3}} \times 100\% \quad \text{(S1)}$$

$$\omega_1 = \frac{\omega_2 \times M_1}{M_2} \quad \text{(S2)}$$

Herein, ρ, *V*, *D*, and *m* respectively represent the mass concentration of DMPP detected by the standard curve (mg/mL), the total volume of sample solution (mL), the dilution times of sample solution during the test, and the weight of the sample (g). ω_1, ω_2, M_1, M_2 is the mass fraction of DMPZ in the sample (%), the mass fraction of DMPP (%), the molar mass of DMPZ (g/mol), and the molar mass of DMPP (g/mol), respectively.

Movie S1. The phosphorous-solubilizing process of P(AA-*co*-AM)-*e*-NI.

Movie S2. The soil-loosening process of P(AA-*co*-AM)-*e*-NI.
